# Multi-Mechanistic In Vitro Evaluation of Antihyperglycemic, Antioxidant and Antiglycation Activities of Three Phenolic-Rich Indian Red Rice Genotypes and In Silico Evaluation of Their Phenolic Metabolites

**DOI:** 10.3390/foods10112818

**Published:** 2021-11-16

**Authors:** Ashrita C. Haldipur, Nagarajan Srividya

**Affiliations:** Department of Food and Nutritional Sciences, Sri Sathya Sai Institute of Higher Learning (Deemed to Be University), Anantapur 515001, Andhra Pradesh, India; ashritachaldipur@sssihl.edu.in

**Keywords:** antidiabetic, AGEs, metabolomics, phenolic profile, molecular docking

## Abstract

The study evaluated the antidiabetic potential of three traditional Indian red rice genotypes/RR (Kattuyanam/KA, Chennangi/CH & Karungkuruvai/KU) using a combination of in vitro, metabolomics (Quadrupole-Time of Flight-Liquid chromatography-Mass spectrometry/Q-TOF-LC-MS/MS)*,* and in silico techniques. In terms of antihyperglycemic potential, KA exhibited the highest inhibitory activity against α-amylase; CH against α-glucosidase; and KU against DPPIV and PTP1B enzymes. KA exhibited the highest antioxidant activity (DPPH, FRAP, and ABTS) and greater inhibition of protein glycation compared to other RR indicating its potential to mitigate diabetic complications. The metabolomic analysis confirmed the presence of 99 phenolics in the sample extracts (KU-71, KA-70, CH-68). Molecular docking studies revealed seven metabolites to be good inhibitors of the four target enzymes and activators of insulin receptor substrate/IRS. The antihyperglycemic and oxidation-glycation reduction composite index revealed KA to have the highest overall antidiabetic potential. Hence, the RR could be utilized in functional foods with a multi-barrelled strategy for diabetes prevention/management.

## 1. Introduction

Diabetes has become a global disease with approximately 463 million people suffering from this metabolic disorder. An estimated increase of 50% i.e., a total of approximately 700 million people are expected to suffer from diabetes by the year 2045 [1]. Hyperglycemia is a major driving force along with other risk factors in the genesis of diabetic symptoms and complications. The consequences of long-term hyperglycemia are alterations in insulin signaling mechanisms and glucose metabolism leading to insulin resistance and beta-cell dysfunction in target organs [2]. Drugs targeting hyperglycemic control through different mechanisms are available in the market these days. However, synthetic antidiabetic drugs may often cause side effects, such as gastrointestinal disturbances, nasopharyngitis, urinary tract infection, myalgias, arthralgias, headache, dizziness, etc. Another problem faced while using synthetic drugs in diabetes treatment is the occurrence of hypoglycemia [3]. Hence, a search for safer treatments from natural sources is gaining importance.

Additionally, in the present global pandemic situation due to COVID-19, diabetes mellitus has become a cause of major concern. This is because patients suffering from COVID-19 and diabetes have exhibited an increased mortality rate compared to other patients without diabetes as a co-morbidity [4]. Hence, the management and prevention of diabetes have further gained a lot of importance.

The focus of functional foods for diabetes has mostly been on herbs, spices, and other food groups that are consumed in fewer quantities as a part of the daily diet [5]. The carbohydrate portion of an average person’s diet is almost 60% or more [6]. However, carbohydrate-rich staple foods such as white rice have been involved in the etiology of diabetes [7]. Therefore, there is an increasing trend to identify healthy carbohydrate-based functional foods for diabetics. Whole grains such as brown rice and other pigmented rice varieties hold promise as a suitable candidate owning to the presence of phytochemicals, dietary fiber, and resistance starch [8]. The suitability of carbohydrate-based foods for diabetic people is generally evaluated using in vitro and in vivo glycemic response studies [9]. To understand the mechanism behind the glycemic response of these rice varieties, the previous studies have focused primarily on the inhibition of carbohydrate metabolizing enzymes.

Other glucose regulatory mechanisms usually evaluated in plants include inhibition of dipeptidyl peptidase IV enzyme (DPP IV) and protein tyrosine phosphatase 1B enzyme (PTP 1B), activation of insulin receptor substrate complex among others [10]. Except for our previous study [11], which assessed the DPP IV inhibitory activity in the extracts of brown Garudan samba and bamboo seed rice, there is a paucity of data in this aspect concerning red rice varieties.

A major problem faced by diabetic people is the development of microvascular (nephropathy, neuropathy, and retinopathy) and macrovascular complications (cardio vascular disease, and cerebrovascular disease) reducing their quality of life greatly [12]. Persistent hyperglycemia leading to the formation of advanced glycated end products (AGEs) is implicated in the development of diabetic complications [13]. Food-derived antioxidants and AGE inhibitors are considered safe and suitable for managing diabetic complications.

The presence of phenolic compounds among other bioactive components are known to contribute to the antihyperglycemic potential of several functional foods [14]. These phenolic metabolites are also known to be good antioxidants and to possess AGEs inhibitory properties [15]. Pigmented rice varieties from different countries such as China, Japan, and Korea have been reported to be rich sources of phenolic compounds [16]. The total phenolic content has been reported totally in approximately ten Indian pigmented rice cultivars from Manipur [17], the Indian Himalayan region [18], and from Tamil Nadu [11,19]. However, metabolomic phenolic profiling has been reported in fewer traditional Indian cultivars [11,20]. Application of metabolomics in diabetes research involves the profiling of metabolites in bioactive plant extracts and the identification of target compounds through approaches such as database and literature screening, as well as in silico analysis with target proteins/enzymes involved in the etiology of diabetes. This is expected to help comprehend the possible mechanisms of anti-diabetic action. Metabolomic phenolic profiling and analysis of several promising traditional pigmented rice varieties remain still largely unexplored.

In our earlier study on eight India pigmented rice varieties, three red rice varieties (Kattuyanam, Chennangi, and Karungkuruvai) were found to have low glycemic index under in vitro conditions [21]. In the current study, the antidiabetic mechanisms in terms of antihyperglycemic, antioxidant, and antiglycation activities of these experimental rice varieties were systematically evaluated using a multi mechanistic combinatorial approach of in vitro, metabolomic, and in silico techniques. 

## 2. Methodology

### 2.1. Experimental Samples 

Three traditional Indian red rice (*Oryza sativa* L.) varieties were selected for this study. The dehusked whole red rice grains (with bran) of Sigappu Kattuyanam (KA) and Karungkuruvai (KU) were collected from organically grown farms in the Theni and Thanjavur districts, respectively in Tamil Nadu, India, and of Chennangi (CH) was purchased from Timbaktu, Dharani organic farm, Andhra Pradesh, India. The morphological details of the samples are presented in Supplementary Table S1. For comparison, a staple white rice variety called Sona masuri (SM) from the Southern parts of India was obtained from a local market in A.P, India. The samples on receipt were sun-dried for approximately 6 h, packed, and stored in the refrigerator until further analysis.

### 2.2. Chemicals and Reagents

From Sigma-Aldrich (Steinheim, Germany) the chemicals—α-amylase (porcine pancreatic), protein tyrosine phosphatase 1B, α-glucosidase (*saccharomyces cerevisiae*), Folin–Ciocalteu’s phenol reagent, gallic acid, quercetin, and ferulic acid-1,2,3-^13^C_3_ and epigallocatechin-2,3,4-^13^C_3_ were purchased. DPP IV inhibitor assay kit was purchased from Cayman Chemicals, Ann Arbor, MI, USA. The solvents were of LC/MS grade (Fisher Chemical, Pittsburg, CA, USA), and all other chemicals from Sisco Research Laboratories, India were of the highest analytical grade. 

### 2.3. Sample Storage and Phenolic Extraction

Required quantities of the experimental samples were precooled at −20 °C and ground to powder using an electric blender. Homogenous samples were obtained by sieving through a 0.5 mm mesh. The samples were freeze-dried in a lyophilizer (Lyodel, Chennai, India), vacuum packaged, and stored at −20 °C until analysis. 

An ultrasonic-assisted phenolic extraction protocol [22] previously standardized in the lab was followed [11]. A solvent mixture (2.5 mL) of methanol: water: formic acid (50: 48.5: 1.5 *v/v*) was added to the freeze dried whole rice powdered samples (0.1 g) and ultrasonicated using Branson ^®^ Ultrasonicator Digital Bath (model no. 5800, Emerson, St. Louis, MO, USA) for 10 min at 50 °C. Centrifugation of the extracts was carried out in a benchtop centrifuge (Sorvall centrifuge, model no. ST 8R, Thermo Fisher Scientific, Lenexa, KS, USA) at 2500× *g* at 5 °C for 5 min. Similarly, the process was repeated for 2 more and the aliquots were combined. The samples extracts were evaporated using a vacuum concentrator (miVac Duo vacuum concentrator, SP scientific, New York, NY, USA) and redissolved in the required quantity of solvents. The extracts were stored at −20 °C until use. 

### 2.4. In Vitro Antihyperglycemic Enzyme Assays

The absorbance and fluorescence measurements in the Section 2.4, Section 2.5 and Section 2.6 were carried out using Varioskan^®^ LUX Multimode Microplate Reader (Thermo Scientific, Lenexa, KS, USA). 

#### 2.4.1. Alpha-Amylase Inhibitory Assay

The α-amylase inhibition assay was carried out as per the method of Visvanathan et al. [23]. The sample extracts (concentrations ranging from 5 to 25 µg/mL) were taken in a 96-well microplate. 40 µL of α-amylase enzyme solution (2 U/mL) and 40 µL of PB (0.02M, pH 6.9) was added and incubated for 10 min at 37 °C. The reaction was started by pipetting 40 µL of soluble starch solution (2 g/L), into the wells and incubated for another 15 min at 37 °C. Finally, 100 µL of glucose oxidase peroxidase (GOD/POD) reagent was added. The absorbance was measured at 505 nm after 15 min. The results were expressed in terms of IC_50_ value. Acarbose, the positive control, was prepared in PBS (2 to 10 µg/mL).

#### 2.4.2. Alpha-Glucosidase Inhibitory Assay

The α-glucosidase inhibitory assay was determined as per the method of Kang et al. [24]. Sample extracts (15 to 100 µg/mL) were added to the microplate wells. To this 112 µL of phosphate buffer (pH 6.8) and 20 µL of α-glucosidase solution (0.2 U/mL in PB) were added. The contents were incubated at 37 °C for 15 min. This was followed by the addition of a 20 µL of 2.5 mM 4-nitrophenyl-β-D-glucopyranosiduronic acid in PB. The reaction was incubated at 37 °C for 15 min and terminated by adding 80 µL of 0.2 M Na_2_CO_3_ solution. The quantity of the *p*-nitrophenol released from PNP-glycoside was quantified by measuring the absorbance at 405 nm. The results were expressed as IC_50_ and acarbose was used as the positive control (5 to 15 µg/mL). 

#### 2.4.3. Dipeptidyl Peptidase IV Inhibitory Assay

The DPP IV inhibitor assay was carried out according to the manufacturer’s protocol and minor modifications [25]. The sample extracts (10 mL) of different concentrations (5 to 25 µg/mL) were taken in a 96-well white-walled microplate. A diluted assay buffer (30 µL) consisting of 20 mM Tris-HCl, pH 8.0, containing 100 mM NaCl, 1 mM EDTA, and 10 µL of diluted DPP IV enzyme (1 ng/mL) was added. 50 µL of diluted substrate solution (3 mM of H-Gly-Pro conjugated to amino-methyl coumarin) was added to initiate the reaction. This was followed by incubation at 37 °C for 30 min. The measurements were taken at 350 to 360 nm excitation and 450 to 465 nm. The results were expressed as a percentage inhibition, IC_50_ and sitagliptin was used as the positive control.

#### 2.4.4. Protein Tyrosine Phosphatase 1 B Inhibitory Assay

The inhibition of the protein tyrosine phosphatase 1 B (PTP 1 B) enzyme was studied according to the method of Fang et al. [26]. To 10 µL of the sample extract/positive control, 20 µL of PTP1B enzyme (1 µg/mL) were added to a microplate. This was followed by the addition of 40 µL of 4 mM p-nitrophenyl phosphate (pNPP) prepared in buffer (25 mM Tris–HCl pH 7.5, 2 mM β-Mercaptoethanol, 1 mM EDTA, and 1 mM DTT). The contents were incubated at 37 °C for 30 min. 30 µL of 2 M NaOH was added to terminate the reaction. The absorbance was measured at 405 nm. During the enzymatic reaction, p-nitrophenyl was produced as a result of pNPP dephosphorylation. The results were expressed as IC_50_ and sodium orthovanadate was used as the positive control.

### 2.5. Antioxidant Assays

#### 2.5.1. DPPH Radical Scavenging Assay 

The 2,2-diphenyl-1-picrylhydrazyl radical scavenging activity was analyzed using the method of Yu et al. [27] Sample aliquots (80–200 μg/mL) were taken and made up to volumes of 40 μL (using 80% methanol). 160 μL of DPPH reagent that was freshly prepared (125 μM in methanol) was then added and vortexed for 10 s. The contents were then incubated in the dark for 30 min at room temperature (37 ± 3 °C). Absorbance was measured after 30 min at 517 nm. The results were expressed as IC_50_ and ascorbic acid was used as the positive control.

#### 2.5.2. FRAP Assay

The ferric reducing antioxidant power (FRAP) was estimated based on the microplate method by Jimenez-Alvarez et al. [28]. To 7.5 µL of the sample extract, 142.5 μL of the FRAP reagent (300 mM acetate buffer at pH 3.6; 10 mM 2,4,6-tripyridyl-s-triazine; 20 mM ferric chloride in the ratio of 10:1:1 respectively) was added. It was incubated for 6 min at room temperature (37 ± 3 °C) and the absorbance was measured at 593 nm. Ferrous sulfate was used as the standard and ascorbic acid was used as the positive control. Results were expressed as Ferrous Equivalent (μmol Fe^2+^/g).

#### 2.5.3. ABTS Assay

The 2,2′-azinobis-(3-ethylbenzothiozoline-6-sulphonic acid) (ABTS) scavenging activity was carried out according to the protocol of Re et al. [29] with modifications to suit the microplate method. The ABTS•+ stock solution was prepared by adding 100 mg of ABTS and 33 mg of potassium persulfate in 50 mL of distilled water (stored overnight in the dark). The working solution was then prepared by diluting approximately 1 mL of the ABTS•+ stock solution with 60 mL of methanol to get an absorbance of 0.70 ± 0.02 at 734 nm. 5 μL of the sample extracts were taken and 200 μL of diluted ABTS•+ solution was added. The absorbance was measured at 734 nm after incubating at a temperature of 30 °C for 6 min. Trolox was used as the standard and butylated hydroxytoluene (BHT) was used as the positive control. Results were expressed as (μmol TE/g). 

### 2.6. Advanced Glycated End Products Inhibitory Potential 

The total protein glycation inhibitory assay using the bovine serum albumin (BSA) model was employed to study in vitro AGEs inhibition [30]. BSA (10 mg/mL) was added to 1.1 M fructose prepared in 0.1 M phosphate buffered-saline (PBS) pH 7.4 containing 0.02% sodium azide. The mixture was incubated in darkness at 37 °C for 3 weeks. Before incubation, 20 µL of the sample phenolic extracts (100 µg/mL phenolic extract dissolved in PBS) were added to the mixture. Glycated BSA was used as a control and 50 mM aminoguanidine was used as a positive control. The formation of fluorescent AGEs was measured by evaluating the fluorescence intensity of glycated BSA [31]. The total AGEs were measured at 350 nm and 440 nm, respectively. The results were expressed as a percentage inhibition.

Inhibition (%) = [1 − ((Fluorescence of samples)/(Fluorescence of control)) × 100]

### 2.7. Total Phenolics and Total Flavonoids Analysis

For total phenolic analysis [32], an aliquot of 20 μL of the sample extract was added to a 96-clear well plate. Freshly prepared Folin- Ciocâlteu reagent (10% *v/v*; 40 μL) was added. After 3 min, 160 μL of 7.5% Na_2_CO_3_ was added. The microplate was incubated for 90 min at 37 °C in darkness. Absorbance was measured at 750 nm. The calibration curve was obtained by preparing a standard solution of gallic acid at concentrations ranging from 5 to 50 μg/mL. The TP content was as gallic acid equivalents in milligrams per 100 g on a dry weight basis (mg GAE/100 g). 

For total flavonoid analysis [33], an aliquot of the sample extract (25 μL) was added into a 96-clear well plate. Double distilled water (125 μL) was added to the extract, followed by the addition of 7.5 μL of 5% NaNO_2_. The mixture was allowed to stand for 5 min, and 10% AlCl_3_ (15 μL) was added. The plate was incubated at ambient temperature for another 5 min, followed by the addition of 1 M NaOH (50 μL). Double distilled water (27.5 μL) was immediately added to dilute the sample mixture and absorbance was read at 510 nm. The calibration curve was made with quercetin as a standard at concentrations ranging from 6 to 50 μg/mL. The TF content was expressed as mg quercetin equivalents (QE) per 100 g on a dry weight basis.

### 2.8. Q-TOF-LC-MS/MS Analysis for Phenolics Identification

The sample extracts were spiked with 30 µL of two internal standards—epigallocatechin-2,3,4-^13^C_3_ and ferulic acid-1,2,3-^13^C_3_ (5 µg/mL) prior to LC-MS analysis. The stable isotopes were added to normalize the data and eliminate variations during ionization and mass analysis. The analytical measurement conditions, data acquisition, treatment, and LC-MS analysis were carried out in the same manner as the method previously standardized in our lab [11]. The instrument used was Quadrupole-Time of Flight-Liquid chromatography-Mass spectrometry/ Q-TOF-LC-MS (Agilent 1290 Infinity LC system, Agilent Technologies, Santa Clara, CA, USA) equipped with a quaternary pump (G4204A), a high-performance autosampler (G4226A), an autosampler thermostat (G1330B) and a column compartment thermostat (G1316C). The system was coupled to an Agilent 6550 iFunnel Quadrupole Time-of-Flight (Q-TOF). 

### 2.9. In Silico Analysis of Red Rice Phenolic Metabolites (RRPM)

#### 2.9.1. ADME Analysis

The ADME (Absorption digestion metabolism and excretion) related parameters of the 20 abundant red rice phenolic metabolites (RRPM) identified through metabolomics analysis were predicted using Swiss ADME (http://www.swissadme.ch/index.php/, accessed on 10 August 2021), a free web-based tool. The RRPM were screened based on Lipinski’s rule (molecular weight <500 Da, lipophilicity (Log P) value less than 5, H-bond donor <5, and H bond acceptor <10) for drug likeliness, GI absorption (predicted using the Boiled-Egg model) and bioavailability score. 

#### 2.9.2. Molecular Docking Analysis

Ten abundant RRPMs obeying Lipinski’s rule, with a good oral availability and bioavailability score were selected as ligands for the study and docked with 5 target proteins (α-amylase, α-glucosidase, DPPIV, PTP1B, insulin receptor substrate) involved in the glucose regulatory pathway using the software AutoDock 1.5.6. The crystalline structures of the target proteins for the study were retrieved from RCSB PDB database (https://www.rcsb.org/, accessed on 10 August 2021)—α-amylase (PDB ID: 2QV4), α-glucosidase, DPPIV (PDB ID: 2I03), PTP1B (PDB ID:4I8N), insulin receptor substrate (PDB ID:1IR3). The protein structures were optimized by removing water molecules and existing complexed inhibitors, followed by the addition of Kollman charges, Gasteiger charges, and the assigning of AD4 type atoms. The 3D structures of the phenolic compounds were obtained from the PubChem database (https://pubchem.ncbi.nlm.nih.gov/, accessed on 10 August 2021) in SDF format which was converted to PDB format using PyMOL (Schrodinger) with the addition of H-bonds.

The active sites of the optimized target proteins were selected depending on the amino acid residues associated with them. The amino acid residues involved in the active sites of the protein were selected based on the literature available: α-amylase—ASP197, GLU233, ASP300 [34]; α-glucosidase—ASP202, GLU271, ASP333, ARG200, HIS105, HIS332, PHE297, TYR65, ASP62, ARG400, PHE166, THR203, PHE206, PHE147, GLY228 [35]; DPP IV—S1 consists of three pockets with different amino acid residues [11,25]; PTP1B—ASN193, PHE280, PHE196, LEU192 RESIDUES, SER187, ALA189, GLY277, CYS215, ARG221, ASP48, GLU115, LEU299, GLU293 [36] and IRS—SER1006, LYS1030, GLU1077, MET1079, ASP1083, ASN1137, and ASP1150 [37]. The spacing of the grid was prepared around the active site of the protein to ensure that ligands fit into the active sites. While performing auto dock, the genetic algorithm was set as the search parameter, other docking parameters were set as default and the output file was obtained in DLG format utilizing a Lamarckian genetic algorithm. Out of the 10 different protein confirmations, the one with the least binding energy (kcal/mol) was selected. PyMOL and BIOVIA discovery studio visualizer software packages were used to visualize the structure of the docked complexes and interpret the types of interactions involved. 

### 2.10. Assessment of Overall Antidiabetic Potential Using AHCI and OGRCI 

To quantitatively evaluate the overall antidiabetic potential of the experimental samples in terms of antihyperglycemic and diabetic complication preventive capacity 2 scoring systems were devised. Antihyperglycemic composite index (AHCI) was based on the inhibitory potential against the 4 enzymes (α-amylase, α-glucosidase, DPP IV, and PTP1B) and the in vitro glycemic index by the authors for the same experimental samples earlier [21]. Equal weights were assigned to all the parameters. First, individual indices were computed for each parameter using the following equation

$$Individual\;index=\frac{100-value\;of\;the\;sample\;}{100-Least\;value\;of\;sample\;or\;positive\;control}\times 100$$

The AHCI was then calculated as the average of the 4 enzyme inhibition score for each sample. 

The oxidation-glycation reduction composite Index (OGRCI) was devised to assess the ability of the experimental rice samples to prevent diabetic complications. It was based on the antioxidant activity (DPPH RSA-% inhibition, FRAP- µmol Fe^2+^/g, TEAC_ABTS_- µmol TE/g) and total glycation inhibition activity (% inhibition) of the samples. First, the sample with the highest value was assigned a score of 100 under each parameter. The indices were then computed for other samples as a percentage of the highest score for each of the parameters.

E.g.,

$$FRAP\;index=\frac{FRAP\;value\;of\;the\;sample}{Highest\;FRAP\;value\;of\;sample\;or\;positive\;control}\times 100$$

The OGRCI score was then calculated as the average of the 4 antioxidant and antiglycation assays. 

An overall antidiabetic potential score was then calculated as an average of AHCI and OGRCI.

### 2.11. Statistical Methods

All the data were mentioned as the mean ± standard deviation of at least 3 replicates. ANOVA analysis (*p ≤* 0.05) with post hoc Tukey’s was carried out to identify significant differences among the samples. Statistical analyses were carried out for all spectrophotometric parameters using SPSS^®^ 21, IBM. Statistical analysis for metabolomic data was conducted using an open-source web platform, MetaboAnalyst 4.0.

## 3. Results and Discussion

The antidiabetic activity of the three red rice extracts, KA, CH, and KU was assessed in terms of their antihyperglycemic activity, antioxidant, and antiglycation capacity under in vitro conditions. This was followed by the phenolic estimation and metabolomic phenolic profiling of the extracts. Further, in silico analysis of the abundant red rice phenolic metabolites was carried out with target proteins involved in the glucose regulatory pathway.

### 3.1. In Vitro Antihyperglycemic Activities of Red Rice Extracts

#### 3.1.1. Alpha-Amylase and Glucosidase Inhibitory Activities

The IC_50_ values for α-amylase inhibition are given in Table 1. KA exhibited the highest inhibition against α-amylase with an IC_50_ value of 2.85 µg/mL similar to the positive control acarbose (2.67 µg/mL). The next higher inhibitory potential was exhibited by KU (IC_50_—4.28 µg/mL) followed by CH (IC_50_—5.71 µg/mL). All three red rice phenolic extracts exhibited significantly lower IC_50_ values than white rice (58.64 µg/mL) indicating better inhibition capacity. The results were in line with findings of another study [38] where the IC_50_ values ranged from 2 to 5.9 µg/mL in other traditional pigmented rice varieties from India. 

The IC_50_ values for α-glucosidase inhibition are given in Table 1. The phenolic extract of CH exhibited the least IC_50_ of 18.51 µg/mL corresponding to the highest α-glucosidase inhibitory potential among the three red rice extracts. Its IC_50_ was only 1.65 times higher than the value for the positive control, acarbose (11.24 µg/mL). The next higher inhibitory potential was exhibited by KA (22.39 µg/mL) followed by KU (24.62 µg/mL) extracts. All the red rice extracts showed significantly higher (*p* ≤ 0.05) inhibitory potential than white rice phenolic extract. 

One of the mechanisms to reduce hyperglycemia is by preventing the breakdown of starch to glucose during digestion by inhibiting target enzymes like α-amylase and α-glucosidase. During the starch digestion process, pancreatic α-amylase is released into the lumen of the intestines where it initiates the process of amylolysis producing the broken down forms, maltotriose, and maltose. The released oligosaccharides are further broken into glucose monomers through the action of α-glucosidase. Inhibition of the two carbohydrate-digesting enzymes results in delaying the digestion as well as the absorption process of starch. Thus, it leads to the suppression of postprandial hyperglycemia [39]. In our earlier analysis of similar samples, α-amylase was strongly correlated with glycemic response [10] and hence, could be recommended as one of the primary methods for evaluating antihyperglycemic activity.

#### 3.1.2. Dipeptidyl Peptidase IV Inhibitory Activity

The phenolic extracts of all the three red rice varieties exhibited DPP IV inhibitory activity with significantly lower IC_50_ values than white rice (Table 1). KU (1.66 µg/mL) showed an IC_50_ value only 1.4 times higher than sitagliptin (1.19 µg/mL), the positive control used in the study. It also exhibited a significantly lower (*p* ≤ 0.05) IC_50_ value than the other two red rice cultivars (2.18–5.68 µg/mL). The DPP IV inhibitory potential of red rice phenolic extracts has been reported for the first time to the best of our knowledge. The results were similar to the values obtained earlier for brown rice extracts—Garudan samba (1.19 µg/mL) and bamboo seed rice (4.22 µg/mL) [11]. Apart from phenolic extracts, other rice components such as rice bran peptides (IC_50_—2.3 mg/mL) and rice grain dipeptides Ile-Pro, Leu-Pro, Val-Pro, and Met-Pro, (IC_50_—1.45 µg/mL) have shown DPP IV inhibitory potential earlier [40,41].

One of the common antihyperglycemic mechanisms observed in diabetic drugs is the inhibition of DPP IV enzyme which prevents the damage of two main glucoregulatory hormones, glucose-dependent insulinotropic polypeptide (GIP) and glucagon-like peptide-1 (GLP-1), thereby maintaining the glucose homeostasis in the body. DPP IV enzyme inhibition is also reported to delay gastric emptying [42].

#### 3.1.3. Protein Tyrosine Phosphatase 1 B Inhibitory Activity

The IC_50_ values for PTP1B inhibition are given in Table 1. The KU phenolic extract exhibited the highest inhibitory potential (IC_50_—30.89 µg/mL) among the three red rice extracts. Its IC_50_ value was only 2.5 µg/mL higher than sodium orthovanadate, the positive control (IC_50_—28.41 µg/mL). The next higher inhibitory potential was exhibited by the methanolic extracts of KA (31.39 µg/mL) followed by CH (38.57 µg/mL). The inhibitory action of white rice extract was not detectable in this study. This is the first study to report the inhibitory effects of red rice phenolics on PTP1B to the best of our knowledge. Barik et al. [43] reported the PTP1B inhibitory effect of methanolic extracts from black and green currants to be IC_50_—11.42 and 19.98 µg/mL, respectively. 

Protein tyrosine phosphatase 1B (PTP1B) is a non-transmembrane phosphatase enzyme, belonging to PTPs class of enzymes and is found in tissues such as liver, muscle, and fat targeted by insulin. It catalyzes the dephosphorylation of the activated insulin receptor, insulin receptor substrate-1, and thereby downregulates insulin signaling. Moreover, it also negatively regulates leptin signaling which causes obesity and metabolic disorders. Thus, inhibition of PTP1B is being considered for the management of type 2 diabetes and the prevention of obesity [44].

### 3.2. In Vitro Antioxidant Activities of Red Rice Extracts

**Figure 1:**
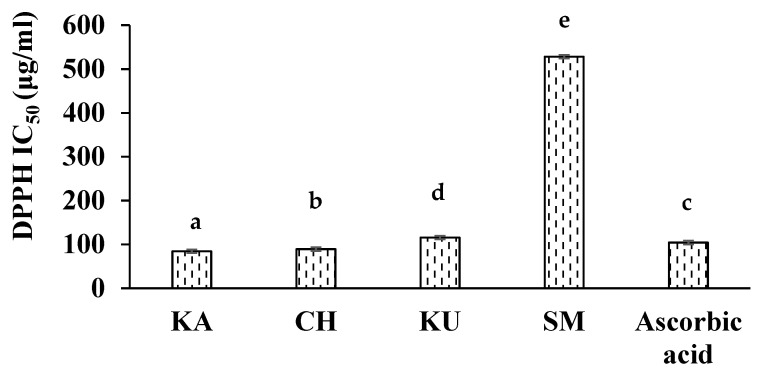
DPPH radical scavenging activity (IC_50_) of the red rice genotypes. KA—Kattuyanam, CH—Chennangi, KU—Karungkuruvai, SM—Sona masuri (white rice). Different alphabets above the columns indicate significant differences across rice varieties at *p* ≤ 0.05.

The antioxidant capacity of food samples with complex matrices is recommended to be evaluated using a minimum of two or three methods [45]. In the present study, the antioxidant potential was evaluated based on the ability to transfer electrons (FRAP) and the ability to transfer both a hydrogen atom and an electron (DPPH RSA and ABTS assay). The antioxidant activities of the phenolic-rich extracts of red rice samples evaluated using the above methods are presented in Table 2 and the DPPH IC_50_ values are shown in Figure 1. 

All the red rice samples exhibited good antioxidant activity and showed a significantly higher (*p* ≤ 0.05) antioxidant potential compared to white rice. The three experimental samples were also found to significantly differ in the activities studied. KA exhibited the highest antioxidant activity in terms of DPPH radical scavenging activity (IC_50_—84.17 µg/mL), FRAP (118.68 µmol Fe^2+^/g), and TEAC_ABTS_ (24.48 µmol TE/g). These values were better or comparable to the respective positive controls (DPPH RSA IC_50_ −104 µg/mL; FRAP—123.27 µmol Fe^2+^/g; TEAC_ABTS_—18.71 µmol TE/g). CH showed the least antioxidant activity in terms of FRAP and TEAC_ABTS_ whereas KU showed the least DPPH RSA. 

The experimental IC_50_ values for DPPH RSA were within the range (50 to 200 µg/mL) reported by Fidrianny et al. [46] for Indonesian pigmented rice genotypes. The experimental FRAP and TEAC values were within the range reported (FRAP—16.54 to 126.19 µmol Fe^2+^/g and TEAC—4.17 to 30 µmol TE/g) for pigmented rice varieties from China [16]. 

Among the three assays, DPPH (*r* = 0.937) and FRAP (*r* = 0.953) assays which showed a high correlation with the total phenolic content could be recommended for antioxidant screening of larger samples of pigmented rice.

Antioxidants-rich red rice extracts are expected to reduce oxidative stress implicated in the occurrence and progression of diabetes and diabetic vascular complications by scavenging free radicals and preventing glycoxidation of proteins which otherwise lead to the formation of advanced glycation end products that are also involved in diabetic complications [47]. 

### 3.3. Advanced Glycation end Products Inhibitory Activity of Red Rice Extracts

The total glycation inhibitory potential of the methanolic extracts of the experimental samples is given in Table 2. The phenolic extracts of KA (68%) and KU (66%) exhibited significantly higher (*p* ≤ 0.05) percentage inhibition than CH (55%). Aminoguanidine, the positive control in the study, exhibited a percentage inhibition of 91%. All three red rice extracts exhibited significantly higher (*p* ≤ 0.05) percent inhibition compared to white rice (5.35%). Daiponmak et al. [48] reported 45 to 70% inhibition of total glycation by pigmented rice varieties from Thailand.

Prolonged hyperglycemia leads to the formation of advanced glycation end products (AGEs) in the body. A non-enzymatic reaction occurs between reducing sugars with free amines of proteins in the body. Glycation is implicated in the pathogenesis of diabetes mellitus as well as in microvascular and macrovascular complications. It is also an important factor in the pathogenesis of other conditions such as atherosclerosis, heart failure, inflammation, rheumatoid arthritis, and neurodegenerative disorders [12]. Hence, the inhibition of AGEs formation is an important mechanism in the prevention and long-term management of diabetes and its complications.

### 3.4. Total Phenolic and Total Flavonoid Contents of Red Rice Extracts

The total phenolic (TP) and total flavonoid (TF) contents of the experimental samples are presented in Table 3. The highest total phenolic content was found in KA (731.41 mg GAE/100 g) followed by KU (510.96 mg GAE/100 g) and CH (499 mg GAE/100 g). The total flavonoid content was also found to be the highest in KA (246.13 mg QE/100 g) followed by CH (243 mg QE/100 g) and KU (235.24 mg QE/100 g). 

The TP and TF values of all the three red rice varieties were significantly higher (*p* ≤ 0.05) than white rice. The TP values of experimental samples were similar to the values (206 to 947 mg/100 g) reported by Deng et al. [16] for pigmented rice varieties from China. The experimental samples exhibited higher TP and similar TF contents compared to red rice varieties (TP—266 to 494 mg GAE/100 g; TF 110 to 236 mg/100 g) from Cambodia, France, and Thailand [49].

Phenolic compounds are secondary metabolites synthesized through the shikimic acid and phenylpropanoid pathways in many foods and plants. Many of them possess several bioactive properties and play a role in combating several chronic lifestyle-related diseases such as diabetes, cardiovascular disease, cancer, etc. [50].

### 3.5. Metabolomics Phenolic Profiling

The retention time (RT), m/z ratio, formula, MS fragments, and scores of the phenolic compounds identified in the experimental samples through metabolomic analysis are given in Supplementary Table S2. The total ion chromatograms of the experimental samples are presented in Supplementary Figure S1. 

Q-TOF-LC-MS/MS analysis revealed the presence of ninety-nine red rice phenolic metabolites (RRPMs) across all three varieties. Flavonoids constituted 65% of the total number of compounds identified. This indicates that flavonoids are the most abundant class of phenolic metabolites present in the red rice varieties. A similar observation was made previously in Indian brown rice varieties, Garudan samba, and bamboo seed rice [11]. The number of subclasses of flavonoids identified included sixteen anthocyanins, thirteen flavonols, ten flavanones, nine flavanols and isoflavonoids, and eight flavones. Dietary flavonoids have been reported for their antidiabetic potential via different mechanisms such as increasing glucose uptake in adipocytes and muscle cells by activating GLUT2 and PPARγ, reducing postprandial blood glucose levels, increasing insulin secretion, and activating insulin receptor [51].

Phenolic acids constituted 26% of the total number of identified metabolites. The number of subclasses of phenolic acids identified in all the samples included fourteen hydroxycinnamic acids, seven hydroxybenzoic acids, three hydroxyphenylacetic acids, and two hydroxyphenylpropanoic acids. Phenolic acids have been reported to possess antihyperglycemic potential through different mechanisms. They have been reported to improve β-cell regeneration, increase insulin sensitivity by activating PPARγ, stimulate first phase insulin secretion by blocking the K^+^ channel in β cells, suppress the expression of advanced glycated end product receptor, normalize hyperglycemia and reverse dyslipidemia [52]. 

Other polyphenols included 9% of the total number of phenolic compounds identified. They included 2-methyl citric acid, sinapaldehyde, 3-methylcatechol, citric acid, ferulaldehyde, 4-hydroxybenzaldehyde, esculetin, mullein, and 4-ethylguaiacol. 

#### 3.5.1. The Unique Phenolic Fingerprint of Red Rice Varieties

The maximum number of seventy-one metabolites were identified in KU, the unique compounds were—1-caffeoylquinic acid, 3-methoxynobiletin, 6-hydroxyluteolin 7-O-rhamnoside, cyanidin 3,5-O-diglucoside, and epigallocatechin. A total of seventy phenolic compounds were identified in KA. The compounds, 1-O-feruloyl-beta-D-glucose, 4-O-methyldelphinidin 3-O-D-glucoside, cyanidin 3-O-(6′′-*p*-coumaroyl-glucoside), and isoxanthohumol were unique to KA. Sixty-eight metabolites were identified in CH of which three were unique i.e., 4′′-O-methylepigallocatechin 3-O-gallate, *p*-coumaroylquinic acid, peonidin 3-O-(6′′-*p*-coumaroyl-glucoside) and epigallocatechin 3′-O-glucuronide. 

Six RRPMs have been identified for the first time in pigmented rice varieties to the best of our knowledge. They include, 3′,4′,7-trihydroxyisoflavan, 3-methoxysinensetin, 3-methylcatechol, 3′-O-methyl-(-)-epicatechin 7-O-glucuronide, 3′-O-methylcatechin, and myricetin. The remaining phenolic metabolites have been reported previously in other brown, red, and black rice cultivars [11,53,54,55,56,57].

#### 3.5.2. Relative Quantification of Red Rice Phenolic Metabolites 

**Figure 2:**
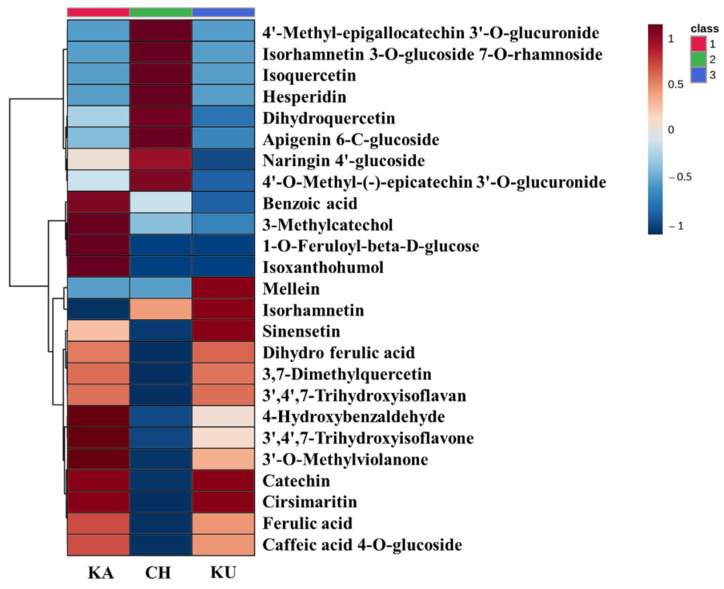
Heatmap showing relative concentrations of top 25 phenolic metabolites in Kattuyanam (KA), Chennangi (CH), and Karungkuruvai (KU).

Hierarchical cluster analysis was carried out using the Ward clustering algorithm and Euclidean distance measure to relatively quantify the top 25 RRPMs out of the total metabolites (83) present in the experimental rice samples. The results are presented in the form of a heatmap (Figure 2) 

The most abundant RRPMs in KA were benzoic acid, 3-methylcatechol, 1-O-feruloyl-beta-D-glucose, isoxanthohumol, 4-hydroxybenzaldehyde, 3′,4′,7-trihydroxyisoflavone, 3′-O-methylviolanone, catechin, and cirsimaritin. In CH, the abundant compounds were found to be 4′-Methyl-epigallocatechin 3′-O-glucuronide, isorhamnetin 3-O-glucoside 7-O-rhamnoside, isoquercetin, hesperidin, dihydroquercetin, apigenin 6-C-glucoside, naringin 4′-glucoside, and 4′-O-Methyl-(-)-epicatechin 3′-O-glucuronide. Five phenolic metabolites catechin, cirsimaritin, mellein, isorhamnetin, and sinensetin were abundant in KU.

#### 3.5.3. Principal Component Analysis of the Red Rice Genotypes with Phenolic Metabolites

**Figure 3:**
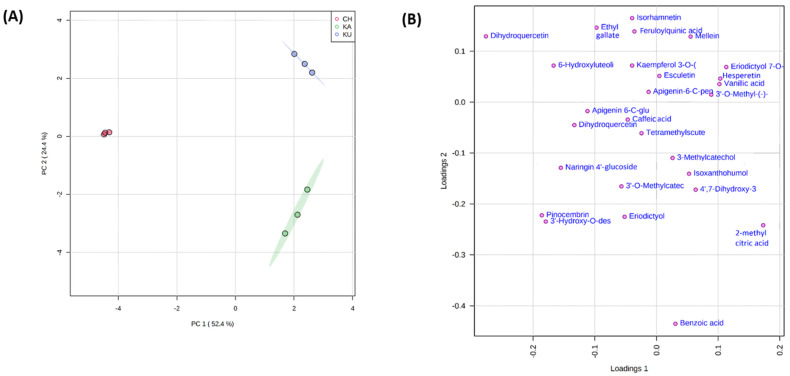
Principal component analysis (PCA) of red rice phenolic metabolites (**A**) PCA score plot (**B**) PCA loading plot.

The principal component analysis (PCA) of the phenolic metabolites resulted in PC1 and PC2 contributing to 76.8% of the total variance. PC1 accounted for 24.2% of the total variance and PC2 accounted for 52.4% of the total variance, which is mainly attributed to the grouping of the red rice genotypes. The score plot and loading plot of the PCA analysis are presented in Figure 3A,B. Interestingly, the PCA plot placed the three experimental samples into three distinguishing groups based on metabolomic phenolic profile.

The red rice genotype, CH was grouped to the top left quadrant of the PCA plot. CH was separated from the other two varieties due to the RRPMs—hesperetin 3′-O-glucuronide, epigallocatechin 3′-O-glucuronide, isoquercetin, 6-hydroxyluteolin 7-O-rhamnoside, and sinapaldehyde. KU was grouped in the upper right quadrant due to the relative concentrations of the RRPMs—epigallocatechin, mellein, 3′-hydroxygenistein, eriodictyol 7-O glucoside, sinensetin, hesperetin, vanillic acid, and 3′-O-methyl-(-)-epicatechin 7-O-glucuronide. Finally, KA was grouped into the right lower quadrant due to the RRPMs—2 methyl citric acid, benzoic acid, 4′,7-dihydroxy-3′-methoxyisoflavan, *p*-hydroxybenzoic acid, isoxanthohumol, 1-O-*p*-coumaroyl-beta-D-glucose, ferulaldehyde, 3-methylcatechol, 4-hydroxybenzaldehyde and 3′,4′,7-trihydroxyisoflavan. The PCA analysis indicated that a different set of phenolic compounds could be contributing to the bioactivities observed in the experimental samples.

#### 3.5.4. Reported In Vitro and In Vivo Antihyperglycemic Activity of the Identified Abundant Red Rice Phenolic Metabolites 

Several in vitro and in vivo studies have reported different antihyperglycemic mechanisms of some of the RRPMs. Yamashita et al. [58] reported isoxanthohumol derived from hop to suppress insulin resistance by modifying the intestinal microbiota and lowering the blood glucose levels in diabetic high fat diet-fed mice. 

Hesperidin has been reported to significantly decrease the fasting blood glucose and pancreatic insulin levels, and pancreatic-duodenal homeobox-1 (PDX-1) protein expression in diabetic mice [59]. Isoquercetin has been reported to reduce hyperglycemia and regulate enzymes of glucose metabolism via insulin signaling pathway in diabetic rats [60]. Samarghandian et al. [61] reported that catechin lowers blood glucose and lipid levels in diabetic rats. A study by Nazir et al. [62] reported that catechin isolated from berries reduced the blood glucose levels and diabetic complication-related biomarkers in streptozotocin-induced diabetic rat models. 

#### 3.5.5. Reported Antioxidant and Antiglycation Activity of Identified Abundant Red Rice Phenolic Metabolites 

Several in vitro and in silico studies have reported the antioxidant activity and glycation inhibitory potential of the abundant RRPMs identified in the studied samples. 

Dhanya et al. [63] reported that hesperidin at 10μM inhibited the non-enzymatic glycation of proteins (65.57%), thereby preventing the formation of advanced glycation end products (AGEs). The study also reported hesperidin to scavenge intracellular reactive oxygen species (ROS) and up-regulate the natural antioxidant defense systems like glutathione under in vitro conditions. 

A study by Potaniec et al. [64] reported the antioxidant activity of isoxanthohumol in terms of DPPH RSA (IC50—8.38 mM). Cirsimaritin has been reported with good antioxidant potential in terms of different assays, such as ABTS, CUPRAC, FRAP, and DPPH [65]. Isorhamnetin has been reported to inhibit methylglyoxal (MGO), a reactive dicarbonyl produced during glucose metabolism and MGO-induced AGEs, and downregulated the expression of the receptor for advanced glycation end products (RAGE) under in vitro conditions [66]. 

Orsini et al. [67] reported apigenin 6-C-glucoside extracted from Caigua fruits to be a more active free radical scavenger than luteolin 8-C-glucoside in terms of DPPH RSA. Apigenin has also been reported to inhibit MGO-adduct AGEs induced oxidative stress and inflammation in endothelial cells (in vitro) by suppressing the ERK/NF-κB signaling pathway started by the AGEs-RAGE interaction and by up-regulating the enzymatic antioxidant defense system [68].

Isoquercetin extracted from *Moringa oleifera* leaf has been reported to exhibit good antioxidant potential by scavenging DPPH free radicals (IC50—5.91 µg/mL) [69]. The same study also reported its antioxidant activity in terms of FRAP (217.68 mmol FeSO4 equivalent/100 g extract) and ABTS (20 mmol TE/100 g extract). Isoquercetin has also been reported to prevent the glycation of hemoglobin (HbA1c) in diabetic rats [60]. A recent study reported catechin with good in vitro antioxidant potential (DPPH RSA and FRAP), and inhibitory potential of AGEs using in vitro and in silico techniques [70]. A recent study by Liu et al. [71] reported that sinensetin could inhibit the glycation of bovine serum albumin.

Hence, the varied phenolic acids and flavonoid metabolites identified in the red rice cultivars could have contributed to their in vitro antihyperglycemic, antioxidant, and antiglycative activities observed in the present study.

### 3.6. In Silico Analysis of Red Rice Phenolic Metabolites

The analysis of previous literature revealed that most of the abundant RRPMs identified in this study have not been evaluated in silico for their inhibitory/activation potential against all the target glucose regulatory proteins selected in the present study. Hence, in an attempt to identify lead natural compounds and to substantiate the in vitro activities observed in the experimental sample extracts, the in silico analysis was performed. 

#### 3.6.1. ADME Screening of Red Rice Phenolic Metabolites 

The twenty abundant RRPMs were subjected to ADME analysis, and the results are represented in Supplementary Table S3. Ten abundant RRPMs (3′,4′,7-trihydroxyisoflavone, 3-methylcatechol, 3′-O-methylviolanone, 4-hydroxybenzaldehyde, benzoic acid, catechin, cirsimaritin, dihydroquercetin, isorhamnetin, and isoxanthohumol) were found to satisfy the Lipinski’s rule (molecular weight < 500 Da, lipophilicity—Log P < 5, H-bond donors < 5, H-bond acceptors < 10), showed good GI absorption and oral bioavailability scores and were taken up for molecular docking analysis. 

#### 3.6.2. Molecular Docking of Red Rice Phenolic Metabolites with Glucose Regulatory Target Proteins

Molecular docking is a computational method used for identifying enzyme inhibitors/receptor activators or blockers. It checks the binding potential of different ligands (phenolic compounds) to the active site of three-dimensional models of biologically relevant target proteins such as enzymes and receptors. The phenolic compound having the least binding energy (ΔG) indicates the best docking result. In the case of enzymes, the least binding energy of the metabolite corresponds to the highest inhibition and in the case of receptors, the least binding energy corresponds to the highest activation of the receptor.

To assess whether the abundant RRPMs identified contributed to the inhibition of the enzymes (α-amylase, α-glucosidase, dipeptidyl peptidase IV/DPPIV, and protein tyrosine phosphatase1B/PTP1), the molecular docking analysis was carried out. Additionally, the molecular docking of the RRPMs was also carried out with the insulin substrate complex to check for its potential insulin-mimetic activity. This is because reduced insulin secretion and insulin resistance have been reported to account for approximately 90% of the different cases of diabetes. In the management of type 1 diabetes and advanced stages of type 2 diabetes, impaired insulin production requires the patients to receive insulin replacement therapy for maintaining their glucose homeostasis [72]. Hence, recent diabetic research is focusing on identifying natural insulin-mimetics agents that can mimic the actions of insulin thereby promoting the uptake of glucose into tissues, activating signal proteins, influencing the expression of genes, and regulating metabolic processes [73]. 

The binding energies of the ten abundant and bioavailable RRPMs with the five target proteins are presented in Table 4. All ten RRPMs showed binding potential with the target proteins. Among the ten RRPMs, seven metabolites (3′,4′,7-trihydroxyisoflavone, 3′-O-methylviolanone, catechin, cirsimaritin, dihydroquercetin, isorhamnetin and isoxanthohumol) exhibited low binding energies (≤−6 kcal/mol) with all five target proteins indicating them to be lead metabolites for antidiabetic potential. 

**Figure 4:**
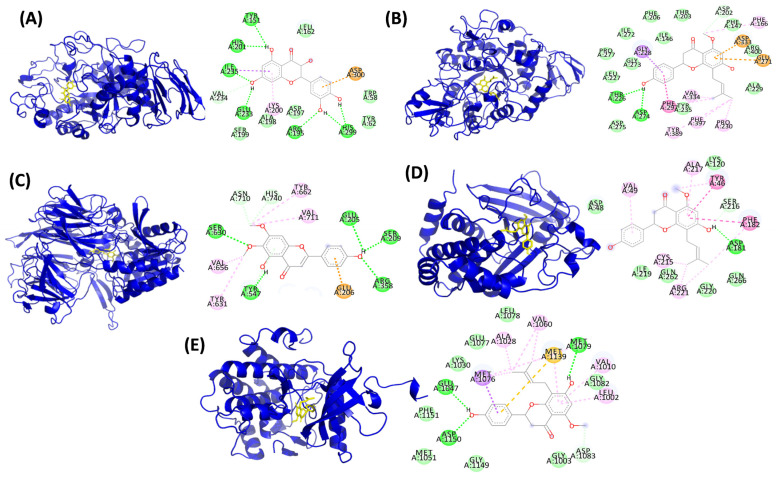
Binding pose and interactions of red rice phenolic metabolites (least binding energy) with target proteins involved in glucose regulation. (**A**) Dihydroquercetin & α-amylase, (**B**) Isoxanthohumol & α-glucosidase, (**C**) Cirsimaritin & DPPIV, (**D**) Isoxanthohumol & PTP1B, (**E**) Isoxanthohumol & insulin receptor substrate.

The molecular interactions between the seven RRPMs (with low binding energies) and the active sites of the five target proteins are summarized in Table 5. The binding poses and molecular interactions of the five RRPMs showing the least binding energy with each of the target proteins are presented in Figure 4.

Among the ten RRPMs, dihydroquercetin exhibited the least binding energy (−7.91 kcal/mol) corresponding to the highest inhibitory potential with respect to α-amylase enzyme. It formed bonds with two active site residues of α-amylase—GLU233 (hydrogen bond) and ASP300 (Pi anion bond). 

In the case of the DPPIV enzyme, cirsimaritin exhibited the least binding energy (−9.79 kcal/mol) and the highest inhibitory potential. It formed bonds with twelve active site amino acid residues—GLU205, SER209, ARG358, TYR547, SER630 (H bonds), ASN710, HIS740 (C-H bond), TYR631, VAL656, TYR662, VAL711 (Alkyl bonds), GLU206 (Pi anion). 

Isoxanthohumol exhibited low binding energy values corresponding to high inhibitory potential with respect to α-glucosidase (−9.26 kcal/mol), and PTP1B enzymes (−8.72 kcal/mol). It formed bonds with seven active site residues of α-glucosidase enzyme i.e., GLU271, ASP333 (Pi anion), GLY228 (Pi sigma), PHE147, ASP202 (C-H bond), PHE166 (Alkyl), PHE297 (Pi-Pi T shaped). It formed bonds with seven active site residues of PTP1B enzyme—ASP181 (H bond), SER216 (C-H bond), ALA217, CYS215, ARG221 (alkyl), TYR46, PHE182 (Pi-Pi T shaped).

Isoxanthohumol also exhibited good binding potential with IRS (−8.42 kcal/mol). With respect to IRS, it formed bonds with three active site residues—MET1079 (H bond), ASP1150 (Pi-sigma), and ASP1083 (C-H bond). 

It is interesting to note that isoxanthohumol, a unique compound abundant in KA, showed very good inhibitory potential against three of the five target proteins and corresponds to the highest in vitro bioactivity of KA observed in the present study. 

Some of the abundant phenolic metabolites identified in the experimental samples have been reported as well in previous in silico studies to exhibit good binding potential at the active sites of different enzymes implicated in diabetes.

Isoquercetin has been reported to bind at the active sites of the enzymes—α-amylase [74], α-glucosidase [75], and DPP IV [76]. Hesperidin has been reported to bind with α-amylase [77], α-glucosidase [78], and dipeptidyl peptidase IV/DPP IV [25] enzymes through molecular docking. Catechin has been reported to show binding potential with α-amylase [79], α-glucosidase [80], DPP IV [81], and protein tyrosine phosphatase 1 B/PTP1B [82] enzymes. 4-Hydroxybenzaldehyde has been reported to bind with α-amylase [11], α-glucosidase [83], DPP IV [11], and PTP1B [84] enzymes.

The results thus indicate the experimental RRPMs identified in the present study to play a role in the inhibition of target diabetic enzymes and the activation of IRS. Several RRPMs such as 3′,4′,7-trihydroxyisoflavone, 3′-O-methylviolanone, and cirsimaritin, to the authors’ knowledge, seem to have been identified for the first time as potential lead natural molecules with multiple glucose regulatory activities.

### 3.7. Overview of the Antidiabetic Potential of the Red Rice Cultivars

**Figure 5:**
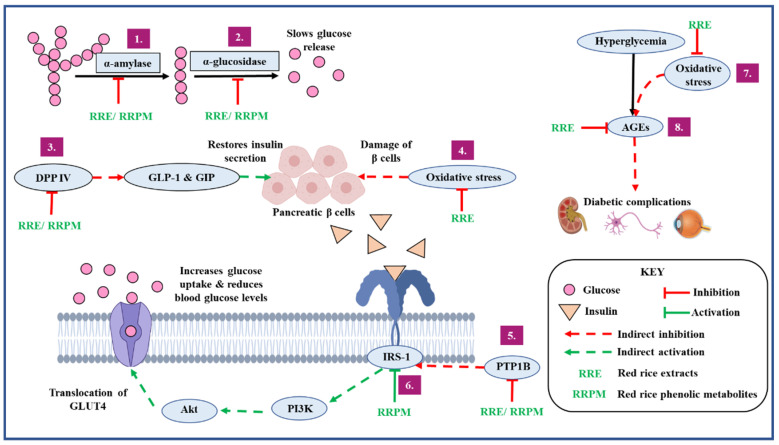
Antidiabetic mechanisms identified in experimental red rice extracts and phenolic metabolites using in vitro and. in silico evaluation.

The present study used varied but complementary techniques reflecting different mechanisms of action for the detailed evaluation to arrive at a holistic conclusion regarding the antidiabetic potential of the experimental red rice samples. To summarize, the experimental red rice samples exhibited the following multiple (eight sites) antidiabetic mechanisms under in vitro and in silico conditions (Figure 5):

- Delaying gastric emptying leading to the reduction of post-prandial blood glucose by inhibiting carbohydrate metabolizing enzymes implicated in diabetes;
- Increasing insulin secretion by preventing the degradation of incretin hormones through DPP IV inhibition;
- Improving insulin sensitivity and insulin signaling by inhibiting PTP1B enzyme;
- Preventing the development and progression of diabetes and its vascular complications by reducing oxidative stress;
- Preventing the development and progression of diabetes and its vascular complications by inhibiting the formation of AGEs;
- Increasing uptake of glucose by activating the insulin receptor complex.

**Figure 6:**
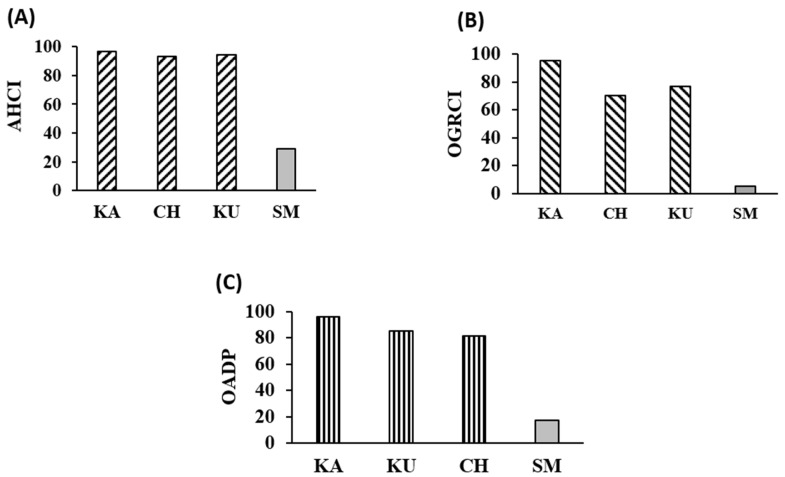
Antidiabetic indices of red rice genotypes. (**A**) Antihyperglycemic composite index (AHCI) (**B**) Oxidation-glycation reduction composite index (OGRCI) (**C**) Overall antidiabetic potential score (OADP). KA—Kattuyanam, CH—Chennangi, KU—Karungkuruvai, SM—Sona Masuri (White rice).

To ascertain the overall antidiabetic capacity of the red rice varieties quantitatively the antihyperglycemic composite index (AHCI) and oxidation-glycation reduction (OGRCI) scores were first computed and are presented in Figure 6A,B, respectively. 

Among the three samples, KA exhibited the highest AHCI score of 96 followed closely by KU (94) and CH (93), whereas white rice exhibited a low AHCI score of 29.

KA also exhibited the highest OGRCI score of 96. KU and CH exhibited relatively lower scores of 77 and 70, respectively. On the contrary, white rice exhibited an exceptionally low OGRCI score of 5.

The overall antidiabetic potential score derived from AHCI and OGRCI (Figure 6C) was highest in KA (96). KU and CH also exhibited good overall antidiabetic scores of 85 and 81, respectively. White rice exhibited a poor score of 17. 

## 4. Conclusions

Among the three experimental samples, Kattuyanam is known for its desirable agronomic traits such as flood & pest resistance, and indigenous knowledge from South Indian villages mentions its traditional use in the dietary management of diabetes. Our study confirms this aspect of the ethnic knowledge by indicating KA to be relatively better than the other two red rice varieties in terms of its overall antidiabetic potency. This scientific evidence could help in boosting the indigenous farming community and economy. The metabolomic phenolic fingerprinting carried out for the first time in the three experimental red rice samples has led to the identification of new metabolites which could be promising lead natural compounds for further phytopharmacological exploration in the field of diabetes. Additionally, new antidiabetic mechanisms such as inhibition of DPP IV and PTP 1B enzymes, and activation of insulin receptor substrate complex by red rice phenolic-rich extracts/metabolites have been identified. Moreover, this is the first study that systematically evaluated the multi-glucose regulatory mechanisms of the three heritage red rice genotypes and ascertained that they could play a role in the dietary management of diabetes and the prevention of diabetic micro and macrovascular complications. A combination of at least two or more bio-mechanisms is recommended for a more effective therapeutic action referred to as a double or multi-barreled approach. Hence, owing to their potential ability to reduce blood glucose levels through multiple strategies, the red rice genotypes evaluated in the current study could be used in the formulation of multi-barreled functional food products for diabetes management. Further studies to evaluate the in vivo glycemic response in humans and the antidiabetic potential of developed functional food products using the experimental samples are under progress. Feeding trials to ascertain the long-term effect on glucose regulatory markers in vivo are needed to further substantiate the observations of the present study.

## Figures and Tables

**Table 1:** Antihyperglycemic potential of the red rice genotypes.

Red Rice Genotypes	α-Amylase IC_50_ (µg/mL)	α-Glucosidase IC_50_ (µg/mL)	DPP IV IC_50_ (µg/mL)	PTP 1B IC_50_ (µg/mL)
Kattuyanam	2.75 ± 0.09 ^a^	22.39 ± 0.08 ^c^	2.18 ± 0.08 ^c^	31.39 ± 0.18 ^c^
Chennangi	5.71 ± 0.07 ^c^	18.51 ± 0.13 ^b^	5.68 ± 0.14 ^d^	38.57 ± 0.21 ^d^
Karungkuruvai	4.28 ± 0.11 ^b^	24.62 ± 0.12 ^d^	1.66 ± 0.06 ^b^	30.89 ± 0.16 ^b^
Sona masuri (White rice)	58.64 ± 1.15 ^d^	82.35 ± 0.19 ^e^	72.57 ± 1.30 ^e^	ND
Positive controls	2.67 ± 0.08 ^a^ (Acarbose)	11.24 ± 0.12 ^a^ (Acarbose)	1.19 ± 0.14 ^a^ (Sitagliptin)	28.41± 0.19 ^a^ (Sodium orthovanadate)

Data are expressed as mean ± standard deviation (*n* = 3). Different alphabets within the same column indicate significant differences across rice varieties at *p* ≤ 0.05. DPP IV—Dipeptidyl peptidase IV, PTP1B—Protein tyrosine phosphatase 1B.

**Table 2:** Antioxidant activity and advanced glycation end products (AGEs) inhibitory potential of the red rice genotypes.

Red Rice Genotypes	DPPH RSA (% Inhibition)	FRAP (µmol Fe^2+^/g)	TEAC_ABTS_ (µmol TE/g)	AGEs Formation Inhibition (% Inhibition)
Kattuyanam	94.89 ± 0.16 ^e^	118.68 ± 0.30 ^d^	24.48 ± 0.21 ^e^	68.17 ± 0.43 ^c^
Chennangi	92.11 ± 0.08 ^d^	99.68 ± 0.18 ^b^	7.56 ± 0.03 ^b^	55.30 ± 0.21 ^b^
Karungkuruvai	86.31 ± 0.21 ^b^	112.65 ± 0.20 ^c^	9.94 ± 0.04 ^c^	65.90 ± 0.79 ^c^
Sona masuri (White rice)	5.21 ± 0.08 ^a^	7.61 ± 0.21 ^a^	0.67 ± 0.03 ^a^	5.35 ± 0.19 ^a^
Positive controls	89.16 ± 0.11 ^c^ (Ascorbic acid)	123.27 ± 0.16 ^e^ (Ascorbic acid)	18.71 ± 0.06 ^d^ (Butylated hydroxytoluene)	91.21 ± 0.81 ^d^ (Aminoguanidine)

Data are expressed as mean ± standard deviation (*n* = 3). DPPH RSA—2,2-diphenyl-1-picrylhydrazyl radical scavenging activity, FRAP—Ferric reducing antioxidant potential, TEAC—Trolox equivalent antioxidant capacity, ABTS—2,2′-azino-bis(3-ethylbenzothiazoline-6-sulfonic acid, AGEs—advanced glycation end products. Different alphabets within the same column indicate significant differences across rice varieties at *p* ≤ 0.05.

**Table 3:** Total phenolics and total flavonoid contents of the red rice genotypes.

Red Rice Genotypes	Total Phenolics (mg GAE/100 g)	Total Flavonoids (mg QE/100 g)
Kattuyanam	731.41 ± 0.25 ^d^	246.13 ± 0.19 ^d^
Chennangi	499.01 ± 0.22 ^b^	243.14 ± 0.55 ^c^
Karungkuruvai	510.96 ± 0.41 ^c^	235.24 ± 0.11 ^b^
Sona masuri (white rice)	92.34 ± 0.56 ^a^	73.88 ± 0.75 ^a^

Data are expressed as mean ± standard deviation (*n* = 3). Different alphabets within the same column indicate significant differences across rice varieties at *p* ≤ 0.05.

**Table 4:** The binding energy of red rice phenolic metabolites with target proteins involved in glucose metabolism.

Red Rice Phenolic Metabolites	Binding Energy (kcal/mol) with Target Proteins				
	α-Amylase	α-Glucosidase	DPP IV	PTP 1B	IRS
3′,4′,7-Trihydroxyisoflavone	−5.27	−6.84	−6.89	−7.89	−5.05
3-methylcatechol	−4.44	−5.43	−5.17	−5.14	−4.33
3′-O-Methylviolanone	−7.13	−7.84	−6.95	−7.31	−7.00
4-Hydroxybenzaldehyde	−4.14	−4.76	−4.30	−4.99	−4.32
Benzoic acid	−4.66	−4.54	−5.00	−4.87	−3.25
Catechin	−6.72	−8.67	−7.81	−7.17	−7.41
Cirsimaritin	−6.41	−7.86	−9.79	−7.46	−8.00
Dihydroquercetin	−7.91	−7.91	−7.43	−6.89	−7.25
Isorhamnetin	−5.62	−7.89	−7.75	−7.65	−6.93
Isoxanthohumol	−7.64	−9.26	−9.49	−8.72	−8.42

**Table 5:** Summary of the molecular interactions between the red rice phenolic metabolites (with low binding energies) and the active sites of the target proteins involved in glucose regulation.

Red Rice Phenolic Metabolites	α-Amylase	α-Glucosidase	DPPIV	PTP1B	IRS
3′,4′,7-Trihydroxyisoflavone	GLU233, ASP197 (H bonds), ASP300 (Pi anion)	ARG400 (H bond), ASP333 (Halogen, Pi-anion, Pi sulfur), PHE147 (Pi sulfur), TYR65 (alkyl), PHE166 (Pi sigma), GLU271 (H bond)	TYR666 (H bond), TYR662 TYR631, PHE357 (Alkyl), SER209, GLU205, GLU206, TYR547 (H bond)	CYS215, ASP181, SER216 (C-H bonds), TYR46 (C-H bond & Pi-alkyl bond), ARG221 (C-H bond & alkyl interaction), GLN262 (halogen (fluorine bond), ILE219, PHE182 (Pi alkyl), ALA217 (Pi-Alkyl and Pi-Sigma)	ASP 1083 (l H bond), MET1079 (H bond), GLU1077 (Halogen fluorine)
3′-O-Methylviolanone	GLU233 (H bond)	GLY228, PHE147, PHE166, ARG400, ASP333, PHE297 (Van der Waals)	TYR631, TRP659, VAL656, VAL711, ASN710, GLU205 (Van der Waal) ARG358, SER630, TYR662 (H bond) TYR547, SER209 (C-H bond) GLU206 (Pi-donor H bond), PHE357 (Pi-alkyl bond)	PHE182, ILE219, GLN262, ARG221 (Van der Waals), ALA217 (Pi donor H bond), CYS215 (Pi alkyl bond), ASP181 (Pi anion)	GLU1077 (Conv. H bond), ASP1083 (Carbon H bond), MET1079 (Pi-alkyl bond)
Catechin	GLU233, ASP197 (H bonds), ASP300 (Pi anion)	GLY228 (H bond), ARG400 (Pi cation), GLU271, PHE147, ASP202, HIS105 (Van der Waals)	GLU205, GLU206, SER209, TYR662, SER630 (H bonds), TYR547 (Pi donor H bond)	PHE182 (Pi alkyl), SER216, CYS215, TYR46 (C-H bond), ILE219 (Alkyl and C-H bond)	ASP1083, MET1079, GLU1077, ASP1150 (Conventional H bonds)
Cirsimaritin	GLU233 (H bond), ASP300 (Pi anion)	HIS105, ASP202, GLY228, ARG400 (H bonds), PHE166, TYR65 (Pi-Pi stacked), PHE297 (Alkyl)	GLU205, SER209, ARG358, TYR547, SER630 (H bonds), ASN710, HIS740 (C-H bond), TYR631, VAL656, TYR662, VAL711 (Alkyl bonds), GLU206 (Pi anion)	ARG221, CYS215 (H bond), (Van der Waals), ASP181 (Pi anion bond), TYR46 (Pi-Pi stacked), ALA217 (Pi alkyl bond)	MET1079 (Conv. H bond), ASP1083 (Carbon H bond)
Dihydroquercetin	GLU233 (H bond), ASP300 (Pi anion)	GLY228 (H bond), PHE147, THR203, GLU271, ASP62 (Van der Waals), ASP202, ARG200, (H bond), ASP333 (Pi anion), TYR65 (Pi-Pi T shaped)	GLU206 (Pi anion), ARG358, TYR631, ASN710 (Van der Waals), GLU205, SER209, SER630, TYR662, TYR666 (H bond), TYR547 (Pi donor H bond), PHE357 (Pi-Pi T shaped)	Nil	GLU1077, MET1079 (Conv. H bond)
Isorhamnetin	GLU233, ASP197 (H bonds)	ASP202, GLY228, ASP333, ARG400 (H bond), TYR65, PHE147, (Pi-Pi T shaped), PHE166 (Pi sigma), HIS105 (Pi alkyl)	GLU205, SER209, GLU206 (H bonds), SER630, TYR547 (Pi-donor bonds), TYR631 (Pi alkyl bond)	SER216 (Van der Waals), ARG221 (H bond), PHE182 (Pi sigma), TYR46 (Pi stacked), CYS215 (Pi-alkyl) ASP181 (Pi-anion)	GLU1077, MET1079 (Conv. H bond), LYS1030 (alkyl)
Isoxanthohumol	ASP300 (H bond), GLU233 (Pi anion)	GLU271, ASP333 (Pi anion), GLY228 (Pi sigma), PHE147, ASP202 (C-H bond), PHE166 (Alkyl), PHE297 (Pi-Pi T shaped)	SER209, TYR662, TYR666 (H bonds), VAL656 (Pi alkyl), GLU206 (Pi anion), TYR547 (Pi donor), PHE357 (Pi-Pi T shaped bond)	ASP181 (H bond), SER216 (C-H bond), ALA217, CYS215, ARG221 (alkyl), TYR46, PHE182 (Pi-Pi T shaped)	MET1079 (Conventional H bond), ASP1150 (Pi-sigma), ASP1083 (Carbon H bond)

## Supplementary Materials

The following are available online at https://www.mdpi.com/article/10.3390/foods10112818/s1, Figure S1: Total ion chromatograms of (A) Kattuyanam (B) Chennangi (C) Karungkuruvai, Table S1: Morphological details of traditional experimental red rice varieties, Table S2: Retention time, m/z ratio, formula, diff, MS fragments and scores of the phenolic compounds identified in the experimental samples, Table S3: ADME properties of the abundant red rice phenolic metabolites.
