# Supplementary material for: Multi-Mechanistic In Vitro Evaluation of Antihyperglycemic, Antioxidant and Antiglycation Activities of Three Phenolic-Rich Indian Red Rice Genotypes and In Silico Evaluation of Their Phenolic Metabolites
Source: Foods. 2021 Nov 16;10(11):2818. doi: 10.3390/foods10112818 (PMC8617766; doi:10.3390/foods10112818)
Supplement: Supplementary file 1 [file foods-10-02818-s001.zip › foods-1412837-SI.pdf]

Supplementary Material

Title: Multi-mechanistic antihyperglycemic assessment of three Indian red rice cultivars: In silico, in vitro and in vivo analyses

Table S1. Morphological details of traditional experimental red rice varieties.

Sample name	Sample code	Rice grains with bran	Bran %	Grain length (mm)	Grain breadth (mm)	Morphological * classification
Kattuyanam	KA	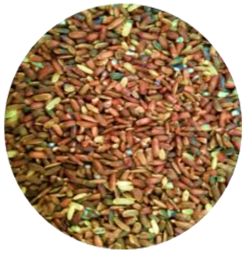	13 %	5.0	2.0	Short & Bold
Chennangi	GS	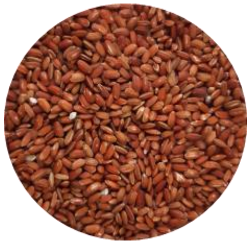	10 %	4.0	2.0	Short & Bold
Karungkuruvai	KU	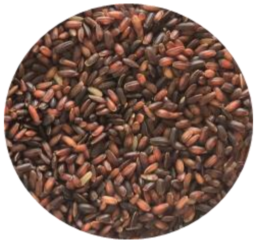	11 %	5.0	3.0	Short & Round

* As per the Food and Agriculture Organization (FAO) of the United Nations, Rome, Italy.

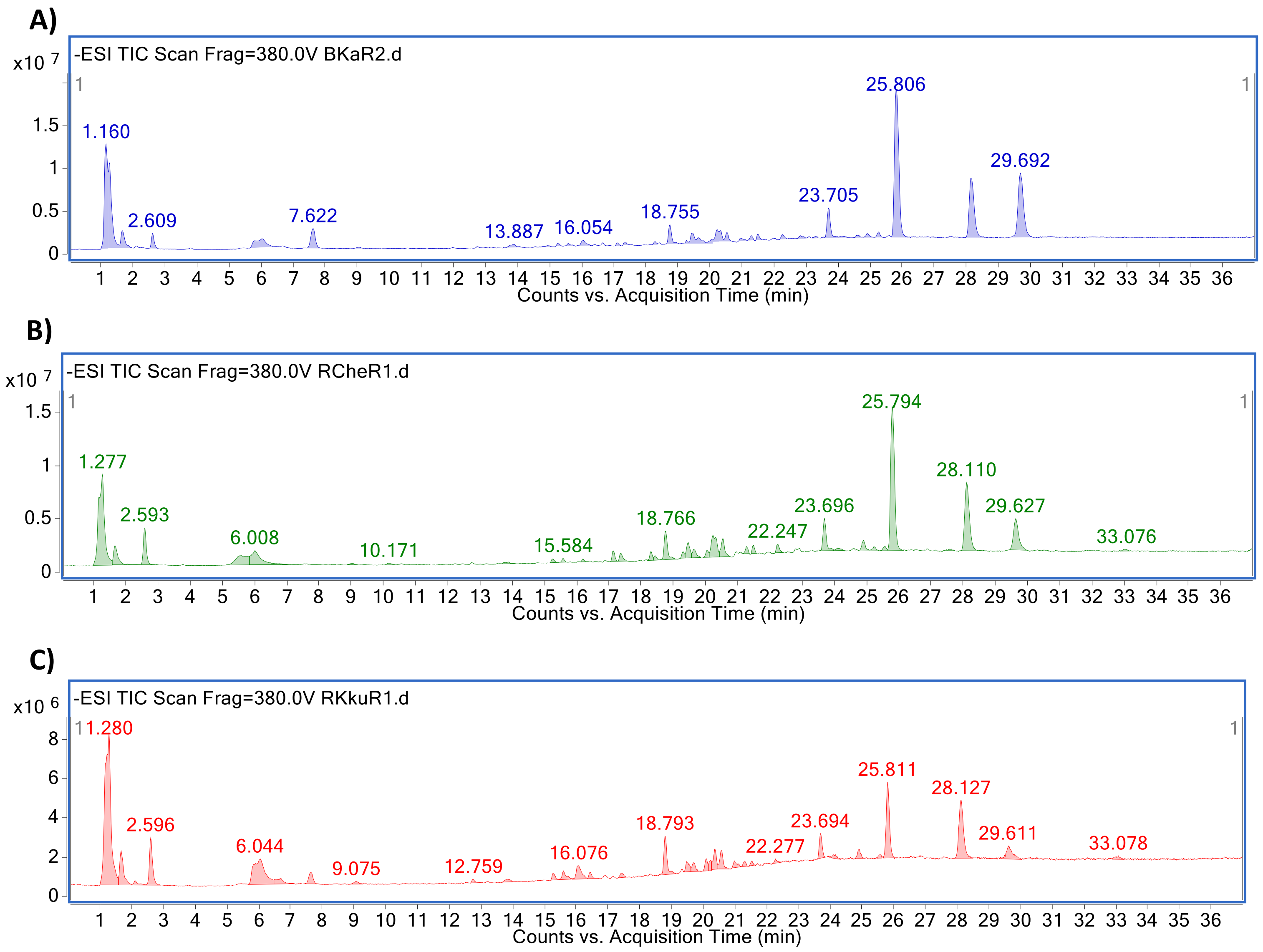

Figure S1. Total ion chromatograms of (**A**) Kattuyanam (**B**) Chennangi (**C**) Karungkuruvai.

Table S2. Retention time, m/z ratio, formula, diff, MS fragments and scores of the phenolic compounds identified in the experimental samples.

S. No.	RT	[M−H]− m/z	Formula	Diff (mDa)	Score	MS fragments	Compound	Class	Sub class
1.	1.105	205.036	$C_7H_{10}O_7$	1.94	85.72	125.0124	*2-methyl citric acid*	*Other polyphenols*	
2.	1.147	479.11895	$C_{22}H_{23}O_{12}$	2.06	84.6	87.0076,123.0440	*4-O-Methyldelphinidin 3-O-D-glucoside*	*Flavonoids*	*Anthocyanins*
3.	1.152	271.0983	$C_{16}H_{16}O_4$	0.55	85.67	225.0557	*3'-O-Methylequol*	*Flavonoids*	*Isoflavonoids*
4.	1.155	480.4188	$C_{22}H_{24}O_{12}$	-4.07	97.25	303.0874, 261.0768, 141.0557	*3'-O-Methyl-(-)-epicatechin 7-O-glucuronide*	*Flavonoids*	*Flavanols*
5.	1.169	304.2946	$C_{16}H_{16}O_6$	-0.86	94.45	149.0244, 123.0451, 111.0451	*3'-O-Methylcatechin*	*Flavonoids*	*Flavanols*
6.	1.205	208.0674	$C_{11}H_{12}O_4$	-0.23	86.94	159.0446, 133.0289	*Sinapaldehyde*	*Other polyphenols*	*Hydroxycinnamaldehydes*
7.	1.222	637.1526	$C_{29}H_{32}O_{16}$	1.41	91.51	325.0348, 269.0449, 147.0293	*Kaempferol 3-O-(6''-acetyl-galactoside) 7-O-rhamnoside*	*Flavonoids*	*Flavonols*
8.	1.231	435.0927	$C_{20}H_{19}O_{11}$	4.32	89.51	101.0238, 115.0395	*Delphinidin 3-O-arabinoside*	Flavonoids	Anthocyanins
9.	1.233	145.0501	$C_9H_6O_2$	-0.36	86.48	129.045	*Coumarin*	*Phenolic acids*	*Hydroxycinnamic acids*
10.	1.238	306.0726	$C_{15}H_{14}O_7$	1.24	87.27	286.0452, 260.0663	*Epigallocatechin*	*Flavonoids*	*Flavanols*
11.	1.282	419.09782	$C_{20}H_{19}O_{10}$	-8.51	87.45	101.0238, 129.0551	*Cyanidin 3-O-arabinoside*	*Flavonoids*	*Anthocyanins*
12.	1.355	134.0143	$C_4H_6O_5$	0.02	98.93	84.9926	*Malic acid*	*Flavonoids*	*Flavonols*
13.	1.705	124.1372	$C_7H_8O_2$	-0.68	87.23	123.0446, 65.0027	*3-Methylcatechol*	*Other polyphenols*	*Alkylphenols*
14.	1.771	167.0342	$C_8H_8O_4$	-0.67	85.07	168.0376, 125.0231	*3,4-Dihydroxyphenylacetic acid*	*Phenolic acids*	*Hydroxyphenylacetic acids*

15.	1.788	167.0334	$C_8H_8O_4$	-1.37	82.26	479.0909, 357.0583	*Vanillic acid*	*Phenolic acids*	*Hydroxybenzoic acids*
16.	1.822	197.0446	$C_9H_{10}O_5$	-0.59	97.71	166.9980, 108.9925, 80.9976	*Ethyl Gallate*	*Phenolic acids*	*Hydroxybenzoic acids*
17.	1.855	179.0337	$C_9H_8O_4$	-1.26	84.57	181.0438, 145.0244	*Caffeic acid*	*Phenolic acids*	*Hydroxycinnamic acids*
18.	1.922	191.019	$C_6H_8O_7$	-0.78	90.56	111.121	*Citric acid*		
19.	2.105	169.0133	$C_7H_6O_5$	-0.97	93.34	169.0218, 126.0337, 124.0225	*Gallic acid*	*Phenolic acids*	*Hydroxybenzoic acids*
20.	2.138	163.0394	$C_9H_8O_3$	-0.57	97.65	163.0395, 119.3241, 93.0354	*p-Coumaric acid*	*Phenolic acids*	*Hydroxycinnamic acids*
21.	2.455	449.1087	$C_{21}H_{22}O_{11}$	-0.13	80.51	285.0399, 149.0238, 107.0133	*Eriodictyol 7-O-glucoside*	*Flavonoids*	*Flavanones*
22.	3.005	343.1078	$C_{19}H_{18}O_6$	-0.99	91.17	285.0762, 199.0395	*Tetramethylscutellarein*	*Flavonoids*	*Flavones*
23.	3.215	178.0629	$C_{10}H_{12}O_3$	0.33	84.63	123.0446, 79.0183	*Ferulaldehyde*	*Other polyphenols*	*Hydroxycinnamaldehydes*
24.	3.805	138.0234	$C_7H_6O_3$	-1	91.13	137.0239, 93.0341	*p-Hydroxybenzoic acid*	*Phenolic acids*	*Hydroxybenzoic acids*
25.	4.005	178.0508	$C_9H_9NO_3$	-0.17	93.02	178.0499, 134.0943	*Hippuric acid*	*Phenolic acids*	*Hydroxybenzoic acids*
26.	4.272	367.1038	$C_{17}H_{20}O_9$	0.29	87.43	173.0449, 143.0344, 59.0133	*Feruloylquinic acid*	*Phenolic acids*	*Hydroxycinnamic acids*
27.	4.555	193.0502	$C_{10}H_{10}O_4$	-0.72	84.6	147.0451, 119.0502	*Ferulic acid*	*Phenolic acids*	*Hydroxycinnamic acids*
28.	5.089	195.0286	$C_9H_8O_5$	-1.27	90.82	147.0082, 121.0289, 107.0133	*Hydroxycaffeic acid*	*Phenolic acids*	*Hydroxycinnamic acids*
29.	5.414	389.1227	$C_{18}H_{18}O_6$	-1.45	92.83	243.0662	*3'-O-Methylviolanone*	*Flavonoids*	*Isoflavonoids*
30.	6.044	595.1451	$C_{30}H_{27}O_{13}$	-2.12	85.24	83.0133, 125.0966	*Cyanidin 3-O-(6''-p-coumaroyl-glucoside)*	*Flavonoids*	*Anthocyanins*
31.	6.289	153.0192	$C_7H_6O_4$	-0.14	99.64	108.9925, 84.9925, 68.9976	*2,4-Dihydroxybenzoic acid*	*Phenolic acids*	*Hydroxybenzoic acids*

32.	6.289	577.1352	$C_{30}H_{26}O_{12}$	0	90.2	578.1407, 425.0862	*Procyanidin dimer B1*	*Flavonoids*	*Anthocyanins*
33.	6.325	595.1663	$C_{27}H_{31}O_{15}$	1.23	88.06	105.0334, 147.0651	*Pelargonidin 3-O-sophoroside*	*Flavonoids*	*Anthocyanins*
34.	6.455	165.0557	$C_9H_{10}O_3$	-0.04	87.61	111.4031, 83.1211	*Dihydro-p-coumaric acid*	*Phenolic acids*	*Hydroxyphenylpropanoic acids*
35.	6.465	315.1135	$C_{15}H_{18}\ O_8$	-0.56	90.23	147.0293, 119.0344, 55.0183	*1-O-p-Coumaroyl-beta-D-glucose*	*Phenolic acids*	*Hydroxycinnamic acids*
36.	6.539	347.0919	$C_{15}H_{18}O_9$	-1.47	90.88	149.0238, 121.0289, 71.0133	*Caffeic acid 4-O-glucoside*	*Phenolic acids*	*Hydroxycinnamic acids*
37.	6.601	355.0652	$C_{15}H_{16}O_{10}$	-1.82	90.3	161.0244, 121.0295	*Caffeic acid 3-O-glucuronide*	*Phenolic acids*	*Hydroxycinnamic acids*
38.	6.672	289.0703	$C_{15}H_{14}O_6$	-1.5	92.29	289.0712, 187.0411, 123.0456	*Catechin*	*Flavonoids*	*Flavanols*
39.	7.272	288.2522	$C_{15}H_{12}O_6$	0.41	89.43	179.0344, 161.0238, 135.0446, 121.0289	*3',4',5,7-Tetrahydroxyisoflavanone*	*Flavonoids*	*Isoflavonoids*
40.	7.372	274.2367	$C_{15}H_{14}O_5$	-0.68	93.94	217.087, 161.0608, 125.0244	*3'-Hydroxy-O-desmethylangolensin*	*Flavonoids*	*Isoflavonoids*
41.	7.572	286.1252	$C_{15}H_{10}O_6$	0.25	92.72	201.0193, 149.0244	*3'-Hydroxygenistein*	*Flavonoids*	*Isoflavonoids*
42.	7.584	121.0289	$C_7H_6O_2$	-0.61	97.34	92.2042, 64.9181	*Benzoic acid*	*Phenolic acids*	*Hydroxybenzoic acids*
43.	7.605	121.0289	$C_7H_6O_2$	-0.64	97.09	121.0294, 108.0218, 93.0345	*4-Hydroxybenzaldehyde*	*Other polyphenols*	*Hydroxybenzaldehydes*
44.	7.672	195.0649	$C_{10}H_{12}O_4$	-1.41	90.72	151.0759, 149.0238, 121.0653	*Dihydroferulic acid*	*Phenolic acids*	*Hydroxyphenylpropanoic acids*
45.	7.739	465.1024	$C_{21}H_{22}O_{12}$	-1.41	90.93	289.0353, 261.0404, 165.0193	*Dihydromyricetin 3-O-rhamnoside*	*Flavonoids*	*Dihydroflavonols*
46.	8.889	563.1408	$C_{26}H_{28}O_{14}$	0.12	99.52	383.2032	*Apigenin-6-C-pentosyl-8-C-hexoside*	*Flavonoids*	*Flavones*

47.	9.389	434.397	$C_{21}H_{22}O_{10}$	1.03	77.66	255.0657, 135.0446, 89.0238	*Naringin 4'-glucoside*	*Flavonoids*	*Flavanones*
48.	9.422	447.0941	$C_{21}H_{20}O_{11}$	0.84	98.04	283.0242, 201.0187, 109.0289, 71.0133	*6-Hydroxyluteolin 7-O-rhamnoside*	*Flavonoids*	*Flavones*
49.	9.789	595.1669	$C_{27}H_{32}O_{15}$	0.25	89.67	287.0555, 135.0082	*Eriocitrin*	*Flavonoids*	*Flavanones*
50.	10.122	303.0517	$C_{15}H_{12}O_{7}$	0.6	98.41	229.0492, 178.9978	*Dihydroquercetin*	*Flavonoids*	*Dihydroflavonols*
51.	10.406	611.1476	$C_{27}H_{31}O_{16}$	1.55	95.06	177.0399, 131.0344	*Cyanidin 3-O-sophoroside*	*Flavonoids*	*Anthocyanins*
52.	10.567	431.0992	$C_{21}H_{20}O_{10}$	0.6	76.39	385.0923	*3-Methoxynobiletin*	*Flavonoids*	*Flavonols*
53.	10.575	433.11347	$C_{21}H_{21}O_{10}$	-3.52	89.94	121.0284, 137.0233	*Isopeonidin 3-O-arabinoside*	*Flavonoids*	*Anthocyanins*
54.	10.806	463.0892	$C_{21}H_{20}O_{12}$	0.93	97.43	301.0388, 243.0328	*Isoquercetin*	*Flavonoids*	*Flavonols*
55.	10.833	482.1004	$C_{21}H_{22}O_{13}$	0.27	98.76	263.0561, 137.0244, 109.0295	*Epigallocatechin 3'-O-glucuronide*	*Flavonoids*	*Flavanols*
56.	10.906	177.0197	$C_{9}H_{6}O_{4}$	0.42	93.19	133.0213, 105.1213, 89.4351	*Esculetin*	*Other polyphenols*	*Hydroxycoumarins*
57.	11.123	740.2155	$C_{33}H_{40}O_{19}$	1.03	83.97	283.0242, 219.086, 135.0082	*Kaempferol 3-O-xylosyl-rutinoside*	*Flavonoids*	*Flavonols*
58.	11.175	403.10291	$C_{20}H_{19}O_{9}$	-3.47	88.25	99.0082, 113.0238	*Pelargonidin 3-O-arabinoside*	*Flavonoids*	*Anthocyanins*
59.	11.356	258.0892	$C_{15}H_{14}O_{4}$	2.38	93.15	119.0502, 109.0295, 93.0345, 77.0396	*3',4',7-Trihydroxyisoflavan*	*Flavonoids*	*Isoflavonoids*
60.	11.506	147.0453	$C_{9}H_{8}O_{2}$	0.08	99.53	147.0446, 145.8880, 102.9346	*Cinnamic acid*	*Phenolic acids*	*Hydroxycinnamic acids*
61.	11.806	624.1627	$C_{28}H_{32}O_{16}$	0.97	97.42	357.0621, 163.0606	*Isorhamnetin 3-O-glucoside 7-O-rhamnoside*	*Flavonoids*	*Flavonols*
62.	12.189	432.1043	$C_{21}H_{20}O_{10}$	0.45	98.95	323.0563, 284.0662	*Apigenin 6-C-glucoside*	*Flavonoids*	*Flavones*

63.	12.189	477.1043	$C_{22}H_{22}O_{12}$	0.45	98.95	151.0036, 109.0295	*Hesperetin 3'-O-glucuronide*	*Flavonoids*	*Flavanones*
64.	12.199	450.1039	$C_{21}H_{22}O_{11}$	0.41	99.51	285.0421, 175.0575, 125.0263	*Dihydroquercetin 3-O-rhamnoside*	*Flavonoids*	*Dihydroflavonols*
65.	12.324	597.1455	$C_{26}H_{29}O_{16}$	0.57	86.28	87.0082, 117.0187	*Delphinidin 3-O-sambubioside*	*Flavonoids*	*Anthocyanins*
66.	12.439	258.2591	$C_{15}H_{12}O_5$	0.18	98.1	163.04, 145.0295	*3',4',7-Trihydroxyisoflavanone*	*Flavonoids*	*Isoflavonoids*
67.	12.506	178.0556	$C_{10}H_{10}O_3$	-0.26	96.87	133.06533, 115.0547, 91.0547	*Mellein*	*Other polyphenols*	*Hydroxycoumarins*
68.	12.748	609.181	$C_{28}H_{34}O_{15}$	-1.15	91.61	260.0623, 224.0495, 150.0315	*Hesperidin*	*Flavonoids*	*Flavanones*
69.	12.856	151.04	$C_8H_8O_3$	-0.08	87	151.0395, 107.0497	*2-Hydroxyphenylacetic acid*	*Phenolic acids*	*Hydroxyphenylacetic acids*
70.	13.198	318.0295	$C_{15}H_{10}O_8$	-0.77	82.96	165.0496, 151.9063	*Myricetin*	*Flavonoids*	*Flavonols*
71.	13.434	602.4335	$C_{40}H_{58}O_4$	1.21	89.67	177.0187, 313.2531	*Gamma oryzanol*	*Phenolic acids*	*Hydroxycinnamic acids*
72.	14.32	609.1608	$C_{31}H_{29}O_{13}$	1.11	88.7	103.0395, 129.0551	*Peonidin 3-O-(6''-p-coumaroyl-glucoside)*	*Flavonoids*	*Anthocyanins*
73.	14.84	301.0357	$C_{15}H_{10}O_7$	0.29	85.24	166.9980, 123.0082	*6-Hydroxyluteolin*	*Flavonoids*	*Flavones*
74.	14.942	402.3946	$C_{22}H_{24}O_9$	-0.99	90.06	386.1247, 284.0833	*3-Methoxysinensetin*	*Flavonoids*	*Flavonols*
75.	14.942	358.1052	$C_{19}H_{18}O_7$	-2.99	80.06	327.0868, 243.0293	*Gardenin B*	*Flavonoids*	*Flavones*
76.	14.973	136.0508	$C_8H_8O_2$	-0.01	86.28	136.04763, 134.89358, 108.0204, 92.0492	*Phenylacetic acid*	*Phenolic acids*	*Hydroxyphenylacetic acids*
77.	15.273	151.0755	$C_9H_{12}O_2$	-0.94	82.67	121.0653, 89.0391	*4-Ethylguaiacol*	*Other polyphenols*	*Alkylmethoxyphenols*
78.	15.35	611.1612	$C_{27}H_{31}O_{16}$	0.65	88.93	103.0395, 131.0344	*Cyanidin 3,5-O-diglucoside*	*Flavonoids*	*Anthocyanins*
79.	15.47	609.18195	$C_{28}H_{33}O_{15}$	0.27	88.19	87.0446, 103.0395	*Peonidin 3-O-rutinoside*	*Flavonoids*	*Anthocyanins*

80.	15.508	271.06	$C_{16}H_{16}O_4$	-1.28	93.68	225.0551, 123.0451	*4',7-Dihydroxy-3'-methoxyisoflavan*	*Flavonoids*	*Isoflavonoids*
81.	15.723	269.0459	$C_{15}H_{10}O_5$	0.38	99.29	224.0496, 135.0076, 119.0129	*3',4',7-Trihydroxyisoflavone*	*Flavonoids*	*Isoflavonoids*
82.	15.746	471.9983	$C_{23}H_{20}O_{11}$	-2	70.7	287.0561, 123.0451	*4''-O-Methylepigallocatechin 3-O-gallate*	*Flavonoids*	*Flavanols*
83.	15.79	301.0718	$C_{16}H_{14}O_6$	0.08	99.19	243.0297, 196.0011, 151.0042	*Hesperetin*	*Flavonoids*	*Flavanones*
84.	15.79	256.0718	$C_{15}H_{12}O_4$	0.08	99.19	213.0554, 151.0037	*Pinocembrin*	*Flavonoids*	*Flavanones*
85.	16.023	329.0673	$C_{17}H_{14}O_7$	0.62	98.55	271.0242, 147.0082	*3,7-Dimethylquercetin*	*Flavonoids*	*Flavonols*
86.	16.03	288.0654	$C_{15}H_{12}O_6$	-1.17	94.69	151.0395, 109.0289, 83.0133	*Eriodictyol*	*Flavonoids*	*Flavanones*
87.	16.03	329.0655	$C_{16}H_{14}O_6$	-1.3	94.93	243.0293, 149.0238, 107.0133, 91.0183	*Homoeriodictyol*	*Flavonoids*	*Flavanones*
88.	16.09	254.0563	$C_{15}H_{10}O_4$	0.25	99.09	534.0665, 508.1121, 254.0539	*7,4'-Dihydroxyflavone*	*Flavonoids*	*Flavones*
89.	16.09	563.1155	$C_{29}H_{24}O_{12}$	-3.9	82.77	517.0770, 129.0551	*Theaflavin*	*Flavonoids*	*Flavanols*
90.	16.64	315.0511	$C_{16}H_{12}O_7$	0.07	86.81	299.0442, 271.9578, 216.9417, 189.0253	*Isorhamnetin*	*Flavonoids*	*Flavonols*
91.	16.076	621.1091	$C_{27}H_{25}O_{17}$	0.59	89.09	109.0284, 135.0076	*Cyanidin 3-O-(3'',6''-O-dimalonyl-glucoside)*	*Flavonoids*	*Anthocyanins*
92.	16.793	463.1240	$C_{22}H_{23}O_{11}$	-4.95	84.57	121.0284, 145.0495	*Isopeonidin 3-O-galactoside*	*Flavonoids*	*Anthocyanins*

93.	16.823	313.0772	$C_{17}H_{14}O_6$	0.01	73.75	313.0712, 243.0293, 173.0238, 67.0183	*Cirsimaritin*	*Flavonoids*	*Flavones*
94.	18.722	356.3246	$C_{16}H_{20}O_9$	0.37	98.01	175.0395, 163.0395, 161.0238	*1-O-feruloyl-beta-D-glucose*	*Phenolic acids*	*Hydroxycinnamic acids*
95.	20.106	496.1217	$C_{22}H_{24}O_{12}$	0.71	89.87	289.0717, 181.0506	*4'-O-Methyl-(-)-epicatechin 3'-O-glucuronide*	*Flavonoids*	*Flavanols*
96.	20.722	353.1427	$C_{21}H_{22}O_5$	3.25	81.6	353.1438, 233.0848	*Isoxanthohumol*	*Flavonoids*	*Flavanones*
97.	20.943	610.15338	$C_{27}H_{30}O_{16}$	-8.26	87.19	101.0238, 119.0344	*Cyanidin 3-O-sophoroside*	*Flavonoids*	*Anthocyanins*
98.	22.027	627.1561	$C_{27}H_{31}O_{17}$	0.26	89.87	89.0238, 161.0449	*Delphinidin 3,5-O-diglucoside*	*Flavonoids*	*Anthocyanins*
99.	22.274	338.1008	$C_{16}H_{18}O_8$	1.51	88.6	221.2218, 191.0575, 163.0392, 119.0504	*p-Coumaroylquinic acid*	*Phenolic acids*	*Hydroxycinnamic acids*

Table S3. ADME properties of the abundant red rice phenolic metabolites.

Molecule	MW (< 500 Da)	LOG P (< 5)	H-bond donors (< 5)	H-bond acceptors (< 10)	Log S	Class	GI absorption	Bioavailability Score
	Lipinski's rule				Water solubility (ESOL)			
1-O-feruloyl-β-D-glucose	356.32	1.68	5	9	-1.64	Very soluble	Low	0.55
3',4',7-Trihydroxyisoflavone	362.32	2.65	1	8	-4.28	Moderately soluble	High	0.55
3-methylcatechol	124.14	0.98	2	2	-2.25	Soluble	High	0.55
3'-O-Methylviolanone	330.33	2.79	1	6	-3.65	Soluble	High	0.55
4-Hydroxybenzaldehyde	122.12	0.99	1	2	-1.87	Very soluble	High	0.55
4'-Methyl-epigallocatechin 3'-O-glucuronide	496.42	1.08	8	13	-2.73	Soluble	Low	0.11
4'-O-Methyl-(-)-epicatechin 3'-O-glucuronide	480.42	1.04	7	12	-2.87	Soluble	Low	0.11
Apigenin 6-C-glucoside	432.38	1.94	7	10	-2.84	Soluble	Low	0.55
Benzoic acid	122.12	1.11	1	2	-2.2	Soluble	High	0.85
Catechin	290.27	1.47	5	6	-2.22	Soluble	High	0.55
Cirsimaritin	314.29	2.56	2	6	-4.2	Moderately soluble	High	0.55
Dihydroquercetin	304.25	1.3	5	7	-2.66	Soluble	High	0.55
Hesperidin	610.56	2.6	8	15	-3.28	Soluble	Low	0.17
Isoquercetin	464.38	2.11	8	12	-3.04	Soluble	Low	0.17
Isorhamnetin	316.26	2.35	4	7	-3.36	Soluble	High	0.55
Isorhamnetin 3-O-glucoside 7-O-rhamnoside	624.54	3.7	9	16	-3.73	Soluble	Low	0.17
Isoxanthohumol	354.4	3.06	2	5	-4.69	Moderately soluble	High	0.55
Mellein	178.18	1.96	1	3	-2.82	Soluble	Low	0.11
Naringin 4'-glucoside	742.68	4.21	11	19	-2.61	Soluble	Low	0.17
Sinensetin	372.37	3.66	0	7	-4.4	Moderately soluble	High	0.55
